# The American Year-Book of Medicine and Surgery

**Published:** 1896-02

**Authors:** 


THE AMERICAN YEAR-BOOK OF MEDICINE AND SURGERY: Being a Yearly Digest of Scientific Progress and Authoritative Opinion in all Branches of Medicine and Surgery, Drawn from Journals, Monographs, and Text-Books of the Leading American and Foreign Authors and Investigators. Collected and arranged with critical editorial comments by J. M. Baldy, M. D., C. H. Burnett, M. D., Archibald Church, M. D., C. F. Clarke, M. D., J. Chalmers Da Costa, M. D., W. A. N. Dorland, M. D., V. P. Gibney, M. D., Homer W. Gibney, M. D., Henry A. Griffin, M. D., John Guitēras, M. D., C. A. Hamann, M. D., H. F. Hansell, M. D., W. A. Hardaway, M. D., T. M. Hardie, B. A., M. B., C. F. Hersman, M. D., B. C. Hirst, M. D., E. Fletcher Ingals, M. D., W. W. Keen, M. D., H. Leffmann, M. D., V. H. Norrie, M. D., H. J. Patrick, M. D., Wm. Pepper, M. D., D. Riesman, M. D., Louis Starr, M. D., Alfred Stengel, M. D., G. N. Stewart, M. D., Thompson S. Westcott, M. D. Under the general editorial charge of George M. Gould, M. D. In one volume of eleven hundred and eighty-three pages. Profusely illustrated with numerous wood cuts in text and thirty-three handsome half-tone and colored plates. Price, cloth, $6.50; half morocco, $7.50. Sold by subscription only. W. B. Saunders, Publisher, 925 Walnut Street, Philadelphia. 1896.

When the early appearance of this work was announced by Mr. Saunders, and it was known that Dr. George M. Gould would edit it, the profession very naturally looked forward to a book of more than ordinary merit. We believe that none will be disappointed in their expectations; in fact, we find the volume even better and certainly much larger and more complete than we had anticipated. It contains nearly twelve hundred royal octavo pages, has thirty-three handsome half-tone and colored plates and numerous wood cuts in the text. The original design of the work, viz.:—To give physicians in a compact form an epitome of the new and progressive medical truths or suggestions published during the months of the preceding year, from July to June, inclusive, has been well carried out. The purpose was not to furnish a literary review of all published matter, but a summary of medical progress; not an epitomization of old knowledge, but only of those things which are or may be contributory to the progress medical science and art. In this way the book will contribute much to the saving of time and advancing the knowledge of the busy physician who wishes to keep up with the march of medical progress, and who can not possibly afford either the expense of buying, or the time to read all of the current medical literature. H.

---

A MANUAL OF DISEASES OF THE EAR FOR THE USE OF STUDENTS AND PRACTITIONERS OF MEDICINE. By Albert H. Buck, M. D., Clinical Professor of the Diseases of the Ear, College of Physicians and Surgeons; Columbia College, New York; Consulting Aural Surgeon, New York Eye and Ear Infirmary, and the Presbyterian Hospital. Second revised edition, one volume, post octavo, 467 pages, illustrated, with blank memoranda pages at back, extra muslin, price, $2.50. William Wood & Company, Publishers, 43, 45 and 47, East Tenth Street, New York City.

In this volume Dr. Buck discusses the various diseases of the internal, middle and outer ear, the auricle, and the mastoid process. The latest devices and appliances for the examination and treatment of the ear, and the proper way to use these appliances, are fully described. Many cases of ear affection are reported from the author's case book, and many valuable deductions are made from these reported cases.

The present edition differs from the one that preceded it in the following points: A new chapter, entitled "Analysis of Symptoms," has been introduced as chapter third; some additional text has been added to the chapter on "Chronic Purulent Inflammations of the Middle Ear," and the section devoted to a description of operations upon the mastoid process has been entirely rewritten and greatly amplified.

The volume contains four hundred and fifty-six pages of closely printed matter, and is one of the best on the subject on which it treats. Dr. Buck's well-known reputation, both as a specialist in this class of diseases and as a teacher, are sufficient of themselves to guarantee the value of this manual. H.

